# Effectiveness of Ultrasonography- and Fluoroscopy-Guided Caudal Epidural Injections in the Management of Chronic Lumbar Disc Disease: A Retrospective Comparative Study

**DOI:** 10.7759/cureus.64844

**Published:** 2024-07-18

**Authors:** Sandeep K Gupt, Ganesh Yadav, Anil K Gupta

**Affiliations:** 1 Physical Medicine and Rehabilitation, King George's Medical University, Lucknow, IND

**Keywords:** lumbar disc disease, low back pain, fluoroscopic, ultrasound, caudal epidural steroid

## Abstract

Introduction: Lumbar disc disease is one of the common causes of lower back pain caused by a change in the structure of the normal disc. Most of the time, disc disease happens as a result of aging and the normal breakdown that occurs within the disc. Caudal epidural steroid injections are the popular treatment for patients with chronic low back pain that does not respond to conservative management.

Method: A retrospective survey was administered to 160 patients who had received caudal epidural injections for chronic low back pain in the past, but only 74 patients who completed the scheduled follow-ups were included in the study. They were divided into two groups based on the imaging modality used for guiding the caudal epidural steroid injections, either ultrasonography or fluoroscopy, and then assessed for improvement in pain on the Numeric Rating Scale (NRS), for functional improvement on the Oswestry Disability Index (ODI), and for satisfaction on the North American Spine Society Patient Satisfaction Scale (SSPSS).

Results: Mean NRS pain scores improved significantly from baseline at 6.78 and 7.00 in the fluoroscopy and ultrasound groups, respectively, to 2.03 and 2.16 at 12 weeks post-procedure. The difference between the groups was not statistically significant (p > 0.05). The Oswestry Disability Index was completed at baseline and after 12 weeks of follow-up for both groups, and there was no significant difference between the two groups; the fluoroscopy group's mean Oswestry Disability Index scores were 52.4 at baseline and 35.6 at 12 weeks, whereas the scores for the ultrasound group were 50.3 at baseline and 37.9 at 12 weeks. Conversely, patient satisfaction as assessed using the SSPSS rose in both groups up to 12 weeks (p > 0.05).

Conclusion: The ultrasound- and fluoroscopy-guided caudal epidural steroid injections proved equally effective in easing the pain, disability, and satisfaction levels of patients with chronic lower back pain.

## Introduction

Lower back pain, particularly when it is chronic, is a common condition affecting many people and accounts for significant disability. Lumbar intervertebral disc disorders are considered to be the most prevalent reason for chronic lower back pain [1]. An interventional technique that is commonly used in the management of chronic low back pain resulting from intervertebral disc disorders is caudal epidural steroid injection [2]. The caudal epidural space provides easy access, allows the injectate to spread to multiple intervertebral spaces, and permits the use of higher volumes and doses compared to other approaches. Caudal epidural injections assisted by fluoroscopy have been in use for several years; fluoroscopy is used not only for precise guidance of the needle but also for documenting the flow of the injectate [3,4]. Although the fluoroscopic images confirm the proper positioning of the needle, they fail to visualize the soft tissue structures. Ultrasonography as a technique has the advantage of providing images of soft tissues and sensitive structures in the spine region and real-time needle tracking [5-7].

Prior systematic reviews have revealed the efficacy of caudal epidural injections in the treatment of pain related to chronic lumbar disc herniation (LDH), but there is a scarcity of evidence pertaining to the use of ultrasonography- or fluoroscopy-guided injections in patients with lumbar disc disease [8]. To achieve interventional pain management success after the caudal epidural injection, it is mandatory to identify and target the sacral hiatus accurately. Both ultrasonographic- and fluoroscopic-guided injections have their pros and cons [9]. Fluoroscopic techniques are invasive because they involve exposure to radiation, but they offer accuracy, whereas CT-guided techniques are invasive and involve radiation exposure but offer better visualization of the joint [10]. Ultrasonography is non-ionizing and less invasive and offers visualization of soft tissues but is highly dependent on the skills of the operator.

Only a handful of trials have compared the effectiveness of ultrasonography-guided caudal epidural injections and fluoroscopy-guided injections [11]. There is no difference between ultrasonography-guided caudal injection and fluoroscopy-guided injection in patients with chronic low back pain due to lumbar disc herniation. To assess the effectiveness of the treatment, the methodology should be sound and the outcome measures must be standardized, both in terms of pain relief and functional improvement and quantitative sensory testing and improvement in the quality of life after the intervention [12]. However, the published studies comparing fluoroscopic and ultrasonographic guidance before the caudal epidural injection do not have adequate sample sizes or methodologically rigorous study designs, so a randomized controlled trial is needed. In addition, long-term studies that involve longer follow-ups of the patients are needed to determine whether one imaging guidance modality provides longer-term improvement to the condition than the other.

Comparing the effectiveness of these modalities will help determine which imaging modality enables the best visualization of these structures and which ensures accurate positioning of the needle in the spinal segments of the patients to produce maximal pain relief and functional improvement. Identifying factors such as duration of pain, MRI findings, and quantitative sensory changes that may predict the adequate outcome of injection would also enable the identification of patients who can benefit most from the treatment [13]. Monitoring for the recurrence of any complications is another research topic that is imperative to consider when assessing the overall risk-benefit ratio of the procedure [14].

Consequently, the goal of this study was to establish the effectiveness of ultrasonography- versus fluoroscopy-guided caudal epidural steroid injections in patients with lumbar intervertebral disc disease in terms of improvement in pain relief, functional disability, and overall satisfaction with the procedure before and after the intervention at predetermined times. Defining the most suitable imaging guidance method can help prevent injury to the patient structure of interest and deliver the injectate to the desired spinal level for the best pain relief and improved function. This study could go a long way in confirming the safety, efficacy, and efficiency of the caudal approach as a targeted, image-guided drug delivery system likely to deliver positive outcomes to a large number of carefully selected chronic discogenic pain sufferers.

## Materials and methods

This was a retrospective survey of 160 patients who had been diagnosed with chronic lower back pain due to intervertebral disc disease based on clinical examination and MRI at a department of physical medicine and rehabilitation of a tertiary care hospital. The patients were divided into two groups based on whether they had undergone fluoroscopy-guided caudal epidural steroid injections or ultrasound-guided caudal epidural steroid injections. Follow-up assessments were recorded at 1, 3, 6, and 12 weeks as per departmental protocol for patients receiving caudal epidural steroids, and the results of the two injection approaches were compared in terms of pain, disability, and patient satisfaction levels at 1, 3, 6, and 12 weeks. A total of 74 patients who satisfied the inclusion and exclusion criteria, with 37 in each group, were included in the study. Patients of both genders were included if they were between 30 and 80 years old, if they had a disease duration from three months to six years, and if they had completed the scheduled follow-ups at 1, 3, 6, and 12 weeks. Patients who did not complete the scheduled follow-up evaluations, who had incomplete data records, or who underwent a surgical intervention during the study period were excluded. Demographic information was collected, including each patient's name, age, sex, and socioeconomic status along with the diagnosis and duration of the disease.

Pain was assessed using the Numeric Rating Scale (NRS) at multiple time points: baseline (NRS0), one week (NRS1), three weeks (NRS3), six weeks (NRS6), and 12 weeks (NRS12). The NRS is a widely used tool for quantifying pain intensity on a scale from 0 to 10, with higher scores indicating greater pain. Disability was assessed using the Oswestry Disability Index (ODI) at the same time points as the NRS. The ODI measures the degree of disability and functional impairment, providing a comprehensive overview of the patient's physical limitations. Higher scores indicate more limitations. Patient satisfaction was evaluated using the North American Spine Society Patient Satisfaction Scale (SSPSS) at 1, 3, 6, and 12 weeks after injection. This scale assesses patients' satisfaction with their treatment and its outcomes, offering insights into the perceived effectiveness and quality of care. By collecting data on these parameters, the study aimed to provide a detailed and multifaceted understanding of the impacts of fluoroscopy-guided (E5764SD-P6, Toshiba) and ultrasound-guided (SONOACE R7, Samsung) caudal epidural injections on pain, disability, and patient satisfaction over time. Statistical analysis was conducted using SPSS software version 21.0 (IBM SPSS Statistics, Armonk, NY). Paired t-tests were used to compare the mean scores of NRS, ODI, and SSPSS at different time points. A p-value of <0.05 was considered statistically significant.

## Results

Patient demographics

Out of the 160 patients, only 74 completed the scheduled follow-ups. Among these, 37 received fluoroscopy-guided caudal epidural injections, and 37 received ultrasound-guided caudal epidural injections. The demographic details are summarized in Table 1.

**Table 1:** Patient Demographics

Parameter	Fluoroscopy Group (n = 37)	Ultrasound Group (n = 37)	Total (n = 74)	P-Value
Mean age (years)	53.6	50.8	52.2	0.046
Male (%)	81.1%	73.0%	77.1%	0.048
Female (%)	18.9%	27.0%	22.9%	0.041
Duration (years)	2.5	2.3	2.4	0.46
Socioeconomic status	3.2	3.1	3.15	0.53

The average age for all the patients was 52.2 years; however, the patients in the fluoroscopy group were slightly older, with an average of 53.6 years as compared to the patients in the ultrasound group with an average age of 50.8 years. The time from the onset of symptoms to treatment was also similar between the two groups at 2.4 years in total: 2.5 years in the fluoroscopy group and 2.3 in the ultrasound group.

Pain assessment

**Figure 1:**
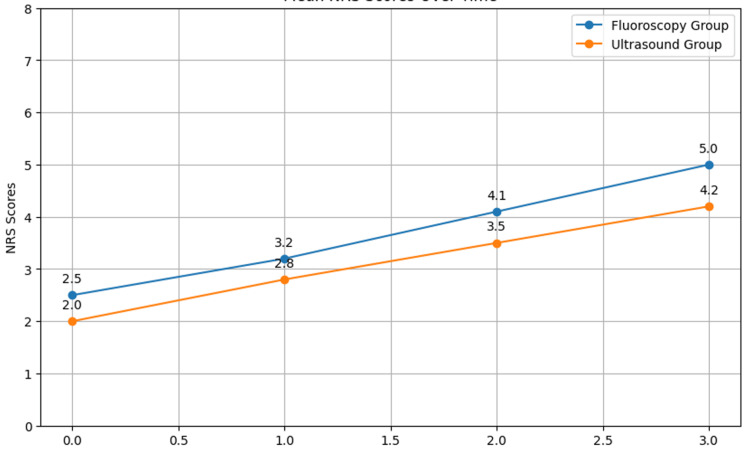
Mean NRS Pain Scores at Subsequent Follow-Up After Caudal Epidural Steroid Injection NRS0: NRS pain scores pre-procedure, with NRS1 at week 1, NRS3 at week 3, NRS6 at week 6, and NRS12 at week 12 post-injection NRS: Numeric Rating Scale

The mean NRS scores at different time points are shown in Table 2 and Figure 1.

**Table 2:** Mean NRS Pain Score A paired t-test was used to compare the mean scores of the NRS at different follow-up times. NRS0: pain scores pre-procedure, with NRS1 at week 1, NRS3 at week 3, NRS6 at week 6, and NRS12 at week 12 post-injection NRS: Numeric Rating Scale

Time Point	Fluoroscopy Group	Ultrasound Group	P-Value
NRS0	6.78	7.00	0.44
NRS1	1.62	1.68	0.82
NRS3	1.41	1.59	0.65
NRS6	1.65	1.81	0.73
NRS12	2.03	2.16	0.76

The Numeric Rating Scale (NRS) measured pain at various time points for the fluoroscopy group and the ultrasound group. At the beginning of the study, the fluoroscopy group had a mean of 6.78 on the NRS0 and the ultrasound group had a slightly higher mean of 7.00 on the NRS0. However, this difference in means was not statistically significant (p = 0.44). Overall, although the numerical differences in the pain scores were revealed between the two groups, the analysis did not expose statistically significant differences at any of the measured time points. Both modalities were equally useful for pain relief from the baseline to the 12-week follow-up period.

Disability assessment

**Figure 2:**
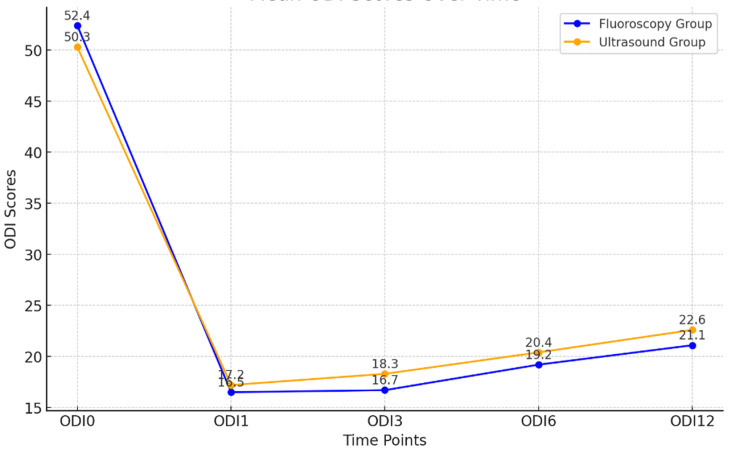
Comparison of ODI Scores at Baseline and Subsequent Follow-Up ODI0: disability scores pre-procedure, with ODI1 at week 1, ODI3 at week 3, ODI6 at week 6, and ODI12 at week 12 ODI: Oswestry Disability Index

The mean ODI scores at different time points are shown in Table 3 and Figure 2.

**Table 3:** Mean ODI Scores at Different Follow-Ups A paired t-test was used to compare the mean ODI scores at different follow-up times. ODI0: disability scores pre-procedure, with ODI1 at week 1, ODI3 at week 3, ODI6 at week 6, and ODI12 at week 12 ODI: Oswestry Disability Index

Time Point	Fluoroscopy Group	Ultrasound Group	P-Value
ODI0	52.4	50.3	0.62
ODI1	16.5	17.2	0.71
ODI3	16.7	18.3	0.58
ODI6	19.2	20.4	0.69
ODI12	21.1	22.6	0.74

Table 3 lists the ODI values of the fluoroscopy group and ultrasound group (p = 0.391) with different periods (baseline, 0, 3, 6, and 12 weeks). The ODI is a self-completed questionnaire for measuring disability in patients with lower back pain, and the score obtained is directly proportional to disability. There was no statistically significant difference between the ODI scores in the fluoroscopy and ultrasound groups at any of the time points post-treatment. This suggests that the changes in disability scores in the two groups that received different imaging-guided treatments for the 12th-week follow-up were comparable.

Patient satisfaction

**Figure 3:**
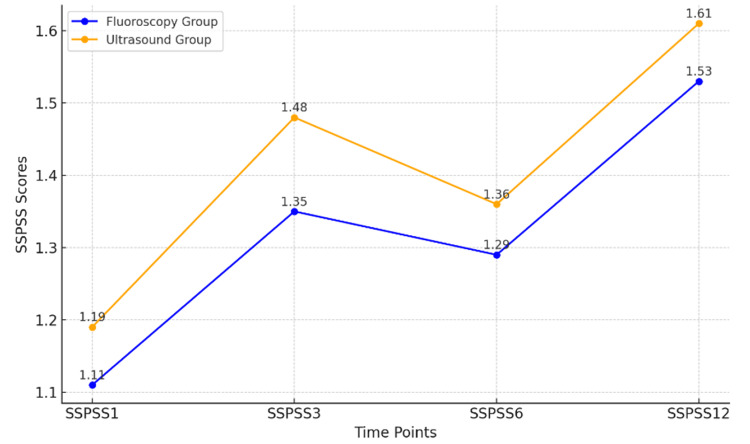
Comparison of SSPSS on Subsequent Follow-Up SSPSS1: satisfaction scores at week 1, with SSPSS3 at week 3, SSPSS6 at week 6, and SSPSS12 at week 12 SSPSS: North American Spine Society Patient Satisfaction Scores

The mean SSPSS scores at different time points are shown in Table 4 and Figure 3.

**Table 4:** Mean SSPSS at Different Follow-Up Times A paired t-test was used to compare the mean SSPSS scores at different follow-up times. SSPSS1: satisfaction scores at week 1, with SSPSS3 at week 3, SSPSS6 at week 6, and SSPSS12 at week 12 SSPSS: North American Spine Society Patient Satisfaction Scores

Time Point	Fluoroscopy Group	Ultrasound Group	P-Value
SSPSS1	1.11	1.19	0.55
SSPSS3	1.35	1.48	0.63
SSPSS6	1.29	1.36	0.72
SSPSS12	1.53	1.61	0.69

The analysis of patient satisfaction, as measured by the North American Spine Society Patient Satisfaction Scores (SSPSS), yielded the following results over various time points. At SSPSS1, the mean score for the fluoroscopy group was 1.11, whereas the ultrasound group had a mean score of 1.19. The p-value of 0.55 indicated no statistically significant difference between the two groups at this time point. The p-value at the final time point was 0.69, which confirmed that there was no statistically significant difference in patient satisfaction between the two groups.

## Discussion

This retrospective study compared the effects of caudal epidural injections via a fluoroscopy-guided technique and an ultrasound-guided technique for managing pain and disability in patients with chronic lower back pain. No significant difference was observed between the two types of imaging at different time points for at least 12 weeks to a year post-injection in terms of pain relief, disability, and patient satisfaction.

Fortunately, there were no significant demographic differences between the two groups. The only notable difference that was observed in the study was that the mean age of the patients in the fluoroscopy group was slightly higher, and the gender ratio was a little skewed, with a few more male patients undergoing fluoroscopy. However, other authors have described the influence of age and gender on the result of epidural steroid injection with less clarity, to the extent that some have denied any impact at all [15,16]. Thus, it can be concluded that these minor differences in the demographic distribution would not have greatly influenced the major outcomes investigated in this research.

The NRS scores indicate that the levels of pain relief provided by the two forms of guidance were similar, which is consistent with previous studies that have noted that the two imaging modalities are equally effective [17]. Fluoroscopy has long been considered the reference method for guidance in injections, but trials have demonstrated that ultrasound may attain similar fluoroscopic accuracy and efficacy [18]. This has been attributed to the fact that ultrasound can capture real-time images of the internal body structures, the progression of the needle, and the dispersion of medication. In addition to the increase in technical accuracy, the application of ultrasound provides other useful benefits such as the elimination of radiation danger, low cost, and the availability of the method [19,20]. In light of these findings, we are inclined to agree with the evolving idea that ultrasound guidance should be considered comparable, if not superior, to fluoroscopic guidance for performing caudal epidural injections.

As expected regarding the pain outcomes, the ODI scores for disability did not reveal any differences between the two study arms through the entire follow-up period of 12 weeks. A previous head-to-head trial that compared ultrasound and fluoroscopy for transforaminal epidural injection also showed no significant difference in terms of ODIs, suggesting that there is no difference in the functional ability between the two groups. This is important because disability significantly relates to how the individual feels the pain; therefore, the similar disability patterns observed in both groups in our study reflect the similar pain relief provided by the allocated interventions.

It could be argued that general satisfaction with the treatment constitutes one of the benefits perceived by patients. The results were quite homogenous in both groups, which ensures that the participants found ultrasound- and fluoroscopy-guided injections beneficial. Earlier researchers have also suggested that patients show similar levels of satisfaction with these two types of imaging procedures [21]. This means that when it comes to acceptability by the patients and the quality of service delivery, none of the modalities is in a better position than the other.

We conclude that the use of ultrasound instead of fluoroscopy can be as effective in administering caudal epidural steroid injections in the treatment of lower back pain in terms of analgesic efficacy, functional status, and patient satisfaction. Some of these radiation-free techniques should be adopted to increase procedural safety while offering comparable therapeutic outcomes, especially in patients having renal disease or deranged renal function and radiation hazards. Subsequent research should examine whether the strategy being investigated for this study has cost benefits, in particular when employing the ultrasound technique.

Limitation

The procedures were performed by one physician, thus reflecting the experience of the practitioner and limiting the generalization of the study results. There is a large number of dropouts and selection bias, so a larger number of cases with a longer follow-up period and a randomized control trial may be needed to overcome the limitations of this study.

## Conclusions

This study revealed no statistically notable differences in pain relief, disability enhancement, or patient satisfaction between patients who underwent fluoroscopic-guided and ultrasound-guided caudal epidural steroid injections for the treatment of chronic lower back pain within a 12-week follow-up period. In addition, there were some small quantitative differences in mean NRS pain scores and mean ODI disability scores between the two groups at multiple follow-up time points, but none of these changes were significant at any point in time. Thus, both modalities were effective at decreasing the pain and disability at follow-up compared to baseline. Because there were no differences between the groups in the key diagnostic characteristics, the results indicate that an ultrasound guide can serve as a less invasive approach for performing caudal epidural injections to improve chronic lower back pain while excluding radiation exposure and offering similar pain relief and functional outcomes.
